# Integrative genomics analysis of genes with biallelic loss and its relation to the expression of mRNA and micro-RNA in esophageal squamous cell carcinoma

**DOI:** 10.1186/s12864-015-1919-0

**Published:** 2015-09-26

**Authors:** Nan Hu, Chaoyu Wang, Robert J. Clifford, Howard H. Yang, Hua Su, Lemin Wang, Yuan Wang, Yi Xu, Ze-Zhong Tang, Ti Ding, Tongwu Zhang, Alisa M. Goldstein, Carol Giffen, Maxwell P. Lee, Philip R. Taylor

**Affiliations:** Genetic Epidemiology Branch, DCEG, NCI, 9609 Medical Center Drive, Rm 6E444 MSC 9769, Bethesda, MD 20892-9769 USA; High-dimension Data Analysis Group, Basic Research Laboratory, Center for Cancer Research, 9609 Medical Center Drive, Rm 1W586, Bethesda, MD 20892 USA; Shanxi Cancer Hospital, Taiyuan, Shanxi 030013 People’s Republic of China; Laboratory of Translational Genomics, DCEG, NCI, Bethesda, MD 20892 USA; Information Management Services, Inc., Silver Spring, Bethesda, MD 20904 USA

**Keywords:** Esophageal squamous cell carcinoma, Biallelic loss, Gene expression, microRNA

## Abstract

**Background:**

Genomic instability plays an important role in human cancers. We previously characterized genomic instability in esophageal squamous cell carcinomas (ESCC) in terms of loss of heterozygosity (LOH) and copy number (CN) changes in tumors. In the current study we focus on biallelic loss and its relation to expression of mRNA and miRNA in ESCC using results from 500K SNP, mRNA, and miRNA arrays in 30 cases from a high-risk region of China.

**Results:**

(i) Biallelic loss was uncommon but when it occurred it exhibited a consistent pattern: only 77 genes (<0.5 %) showed biallelic loss in at least 10 % of ESCC samples, but nearly all of these genes were concentrated on just four chromosomal arms (ie, 42 genes on 3p, 14 genes on 9p, 10 genes on 5q, and seven genes on 4p). (ii) Biallelic loss was associated with lower mRNA expression: 52 of the 77 genes also had RNA expression data, and 41 (79 %) showed lower expression levels in cases with biallelic loss compared to those without. (iii) The relation of biallelic loss to miRNA expression was less clear but appeared to favor higher miRNA levels: of 60 miRNA-target gene pairs, 34 pairs (57 %) had higher miRNA expression with biallelic loss than without, while 26 pairs (43 %) had lower miRNA expression. (iv) Finally, the effect of biallelic loss on the relation between miRNA and mRNA expression was complex. Biallelic loss was most commonly associated with a pattern of elevated miRNA and reduced mRNA (43 %), but a pattern of both reduced miRNA and mRNA was also common (35 %).

**Conclusion:**

Our results indicate that biallelic loss in ESCC is uncommon, but when it occurs it is localized to a few specific chromosome regions and is associated with reduced mRNA expression of affected genes. The effect of biallelic loss on miRNA expression and on the relation between miRNA and mRNA expressions was complex.

**Electronic supplementary material:**

The online version of this article (doi:10.1186/s12864-015-1919-0) contains supplementary material, which is available to authorized users.

## Background

Esophageal cancer is the eighth most common and the sixth most frequent fatal human cancer in the world [1] and the fourth most common incident cancer in China [2]. Shanxi Province, a region in north central China, has among the highest esophageal cancer rates in China and nearly all of these cases are esophageal squamous cell carcinoma (ESCC). ESCC is an aggressive tumor which is typically diagnosed only after the onset of symptoms when prognosis is very poor. The 19 % 5-year survival rate is fourth worst among all cancers in the USA [3]. One promising strategy to reduce ESCC mortality is early detection. Further, a better understanding of the molecular mechanisms underlying esophageal carcinogenesis and its molecular pathology will facilitate the development of biomarkers for early detection.

Genomic instability is one of several mechanisms that can lead to gene dysregulation and has been thought to play an important role in the etiology of human cancers, including both histologic types of esophageal cancer, esophageal adenocarcinoma and ESCC [4, 5]. In previous studies using a variety of different methods we found that LOH was common in ESCCs from Shanxi Province in north central China. These studies identified over 20 common LOH regions, frequent copy number alterations (both gain and loss), as well as numerous copy number neutral regions, which suggests that this cancer is characterized by genomic instability [5, 6]. In addition, somatic mutations in several genes with critical roles in carcinogenesis (e.g., *TP53, CDKN2A,* and *BRCA2*) have been identified in ESCC patients with LOH in regions that include the genes [7–9], indicating that a wide variety of DNA alterations in numerous genes occur in the development of this tumor.

Gene expression microarray technology is an important tool for evaluating tumor heterogeneity and has been successfully applied to identify subsets of tumors (including within ESCC) with different clinical parameters such as survival, histological grade, invasive status, and response to therapy [10–15]. In recent years, miRNAs have emerged as a major class of regulatory genes, and one class of miRNAs, conserved miRNA, has targets which can now be predicted with confidence. It is thought that the role of miRNAs is to control expression of target genes. Thus, dysregulation of miRNA is expected in human diseases such as cancer, which are attributed to dysregulation of gene expression in tumor suppressors and oncogenes [16–18]. Further, dysregulation of some miRNAs has been related to patient survival in some cancers, including ESCC [19, 20]. Biallelic loss is thought to play a critical role in tumor pathogenesis, especially because of its influence on expression of affected genes (mRNA) and related miRNA, however, these relations have not been well studied in ESCC. Since cancer is a complex disease, it is increasingly important that analyses combine the evaluation of alterations in DNA and RNA, including those that occur in both mRNA and miRNA, in order to better understand their potential interactions in the development of cancers.

It has become clear that human genetic variation ranges from single nucleotide changes at the sequence level up to multi-megabase chromosomal aberrations. Of the molecular genetic changes that occur during the development of human cancer, alterations in SNPs are likely among the earliest or even the initial events that lead to genomic instability. While studying large changes (eg, big deletions, inversions, and translocations at the chromosomal level) in tumor cells is informative, knowledge of the more numerous small alterations that occur at the nucleotide sequence level are equally or more critical to our understanding of the detailed process of carcinogenesis, particularly at its earliest stages. For example, biallelic loss in genes may cause double strand breaks which result in widespread structural rearrangements of the genome. However, how alterations of DNA (i.e., biallelic loss) influence gene expression remains largely unknown, especial for SNPs that are not in coding regions.

In the present study, we performed global profiling of alterations in DNA as well as expression of mRNA and miRNA in tumors and their matched normal tissues from 30 ESCC cases. Using these profiles, we identified genes with biallelic loss and examined their mRNA expression and miRNA targets as an initial step in understanding relations among these small alterations in nucleic acids in ESCC.

## Methods

### Case selection

This study was approved by the Institutional Review Boards of the Shanxi Cancer Hospital and the US National Cancer Institute (NCI). Briefly, cases diagnosed with ESCC between 1998 and 2001 in the Shanxi Cancer Hospital in Taiyuan, Shanxi Province, PR China, and considered candidates for curative surgical resection were identified and recruited to participate in this study after obtaining written informed consent. None of the cases had prior therapy and Shanxi was the ancestral home for all. Cases ranged in age from 39 to 67 years (median 56 years) and were predominantly female (63 %). Clinically, most cases had Stage 2 (77 %) cancers and half had evidence of metastasis at diagnosis. The ESCC cases studied here were previously evaluated for LOH and copy number alterations using genome-wide arrays [5, 6].

### Biological specimen collection and processing

Venous blood (10 ml) was taken from each case prior to surgery and germline DNA from whole blood was extracted and purified using the standard phenol/chloroform method. Tumor and adjacent normal tissues were dissected at the time of surgery and stored in liquid nitrogen until used. DNA was extracted from micro-dissected tumor as previously described [5] using the protocol from the Puregene DNA Purification Tissue Kit (Gentra Systems, Inc., Minneapolis, MN).

RNA was extracted from 17 of the micro-dissected tumors and their matched normal tissue pairs as described previously using the protocol from the PureLink Micro-to-Midi Total RNA Purification System (Catalog number 12183–018, Invitrogen, Carlsbad, CA) [5]; total RNA from 13 cases was isolated by using the Allprep kit (Qiagen) per the manufacturer’s instructions. RNA quality and quantity were determined using the RNA 6000 Labchip/Agilent 2100 Bioanalyzer (Agilent Technologies, Germantown, MD).

### Target preparation for GeneChip human mapping 500K array set

The Affymetrix GeneChip Human Mapping 500K array set was previously performed in these patients (6, 7). The set array contains ~262,000 (Nsp I array) and ~238,000 (Sty I array) SNPs (mean probe spacing = 5.8Kb, mean heterozygosity = 27 %). A detailed gene chip protocol can be found at http://www.affymetrix.com/support/downloads/manuals/500k_assay_manual.pdf.

Experiments were conducted according to the protocol (GeneChip Mapping Assay manual) supplied by Affymetrix, Inc. (Santa Clara, CA). Genotype calls were generated by GTYPE v 4.0 software (Affymetrix). Paired germ-line and tumor DNA from each case were run together in parallel in the same experiment (ie, same batch, same day). The GEO accession number for these SNP array data is GSE15526.

### Probe preparation and hybridization for Human Genome U133A 2.0 array

The Affymetrix Human Genome U133A 2.0 array is a single array used to interrogate expression of 14,500 well-characterized human genes. Array experiments were performed using 1-5ug total RNA for each array as described previously [10]. We followed the protocol provided by the manufacturer to carry out reverse transcription, labeling, and hybridization. (http://www.affymetrix.com/support/technical/manual/expression_manual.affx). RNA from paired tumor and normal esophageal tissues were run together in parallel in the same experiment. The GEO accession number for these expression array data is GSE38129.

### ABI miRNA expression array by RT-PCR

The TaqMan® Low Density Array was used to determine microRNA expression in this study, which employed the 9700HT fast real-time PCR system from ABI. Comprehensive coverage of Sanger miRBase v14 was enabled via a two-card set of TaqMan® Array MicroRNA Cards (Cards A and B) for a total of 754 assays specific to 664 unique human miRNAs. In addition, each card contains one selected endogenous control assay (MammU6; printed four times), five human endogenous controls (RNU 6B, 24, 43, 44, 48) that are the most highly abundant and stably expressed across all tissues, and one negative control assay (ath-miR159a). Card A focused on more highly characterized miRNAs, while Card B contained more recently discovered miRNAs along with the miR* sequences.

RNA from paired tumor and normal esophageal tissues were run together in parallel in the same experiment. The protocol followed the manufacturer’s manual at http://www3.appliedbiosystems.com/cms/groups/mcb_support/documents/generaldocuments/cms_042167.pdf. Briefly, three uL of total RNA (350-1000ng) was added to 4.50uL of RT reaction mix, which consisted of 10x Megaplex RT Primers, 100mM dNTPs with dTTP, 50U/uL MultiScribe Reverse Transcriptase, 10x RT buffer, 25mM MgCl^2^, 20U/uL RNase Inhibitor, and nuclease-free H_2_O. The samples were run on a thermal cycler using the following conditions: 40 cycles of 16 °C for two min, 42 °C for one min, and 50 °C for one sec. All reactions were completed with a final incubation of 85 °C for five min. Six uL of cDNA generated from the thermal cycler was mixed with 450uL of 2x TaqMan Universal PCR Master Mix with no AmpErase UNG, and 444uL of nuclease-free H_2_O. 100uL of the reaction mix was added to each of eight fill ports on the TaqMan MicroRNA Array. The filled Array was centrifuged twice at 1200 rpm for one min, and then sealed with the eight fill ports removed. The array was run on the 7900HT RT-PCR System with SDS software. The comparative CT method was used to determine the expression levels of mature miRNAs. The GEO accession number for these miRNA data is GSE66274.

### GeneChip 500K array data analysis

Probe intensity data from Affymetrix 500K SNP arrays were used to identify DNA alterations in the present study. To avoid gender-related issues, SNPs mapped to either the X or Y chromosome were excluded. Affymetrix SNP array data were first normalized using the gtype-probe set-genotype package included in Affymetrix Power Tools version 1.85. Each tumor sample was individually normalized via the BRLMM algorithm along with 99 blood samples. These blood samples were obtained from the 30 ESCC cases evaluated in the present study plus 69 healthy controls (age-, sex-, and region-matched to the cases) who were all part of a larger case–control study of upper gastrointestinal (UGI) cancers conducted in Shanxi Province [21]. Biallelic loss, including loss of both alleles in heterozygotes as well as homozygous deletions, was determined based on comparison of matched tumor versus germline DNA. Several criteria were used to determine biallelic loss as follows: 1) a SNP with biallelic loss must have (a) a “No Call” genotype call in the tumor sample; (b) a high quality genotype call in the normal sample; and (c) reduced copy number (CN0 or CN1); 2) analysis was limited to SNPs in genes (exons and introns only); and 3) analysis was limited to SNPs that fulfilled elements from criterion #1 (a) to (c) in at least 10 % of the 30 ESCC cases studied. Analyses of LOH and CN were described previously [5, 6].

### Human genome U133A 2.0 array data analysis and relation between biallelic loss and mRNA expression

For all of the Affymetrix U133 array data, raw data sets (CEL files on all samples) after scanning were normalized using RMA as implemented in Bioconductor in R (http://www.bioconductor.org), including background correction and normalization across all samples. For each sample, log2 fold changes in gene expression were calculated by subtracting the adjacent normal RMA value from the corresponding tumor RMA value.

To assess the influence of biallelic loss on expression, we performed the following steps: (i) First, genes assayed by the U133A microarray were mapped onto each biallelic loss segment of each sample. Map locations of genes were taken from the Affymetrix version *na29* microarray annotation file. (ii) We then performed two-sided unpaired Wilcoxon rank sum tests comparing the log2 fold changes for a probe set in biallelic loss positive and negative samples. A *P*-value <0.05 was considered significant. (iii) Finally, SNPs on the 500K microarray were mapped to the reference sequence for each expression probe set. Average fold changes were used to relate mRNA expression to DNA biallelic loss.

### ABI miRNA expression array analysis

RQ Manager integrated in software from ABI was used to normalize the entire signal generated. Expression level (as fold change) was calculated when both tumor and normal samples had signals in the assays using DataAssist software v2.0 (Life Technologies, http://www.lifetechnologies.com/about-life-technologies.html). Signals for miRNA that showed either in tumor only or normal only were dropped from analysis. Fold change was calculated using the 2 ^-ΔΔCT^ method. In the present study, the data are presented as fold change in the target gene expression in tumors normalized to the internal control gene (MammU6) and relative to the normal tissue control (matched normal as calibrator). Results of the real-time PCR data are represented as C_T_ values, with C_T_ defined as the threshold cycle number of PCRs at which amplified product was first detected. The average C_T_ was calculated for both the target gene and MammU6 and the ΔC_T_ was determined as (the mean of up to three C_T_ values for the target gene) minus (the mean of the C_T_ values for U6). The ΔΔC_T_ represented the difference between the paired tissue samples, as calculated by the formula ΔΔC_T_ **=** (ΔC_T_ of tumor - ΔC_T_ of normal). The N-fold differential expression in the target gene of a tumor sample compared to its normal sample counterpart was expressed as 2 ^-ΔΔCT^. For each case, the frequency of dysregulated miRNAs was calculated as the number of dysregulated miRNAs divided by the total number of miRNAs that showed signals in both tumor and normal. The criteria used to call an miRNA dysregulated were fold changes ≥ 2 or ≤ 0.5.

We used TargetScan (http://www.targetscan.org/) (Whitehead Institute for Biomedical Research, Cambridge, MA, USA) and Sanger miRBase (http://www.mirbase.org/) to identify conserved miRNAs in the 3′ UTR for affected genes, which are thought to be preferentially conserved.

We used median fold change for both miRNA and mRNA in our analysis of the relation between expressions of miRNA and genes. Correlations and *p*-values between selected variables were performed using Spearman rank correlations and Wilcoxon rank tests.

## Results

A flow diagram detailing the various laboratory analyses performed in the study can be found in Additional file 1: Figure S1.

### Genes with frequent biallelic loss

The overall average genotype call rate was 95 % in the present study for the 60 chips evaluated: average call rates for the 250K Nsp chip were 95 % for both germline DNA (range 93–98 %) and tumor DNA (range 91–97 %), and average call rates for the 250K Sty chip were 96 % (range 90–98 %) for germline DNA and 95 % (range 92–97 %) for tumor DNA.

We identified 702 SNPs that showed frequent biallelic loss, that is, in at least 10 % (at least three cases) of ESCC tumors (see “Methods” section). Those 702 SNPs mapped to 77 genes and represent 9.4 % of the total of 7484 SNPs in those genes on our SNP array. Nearly all of the 77 genes represented by these SNPs were concentrated on just four chromosomal arms (ie, 42 genes on 3p, 14 on 9p, 10 on 5q, and 7 on 4p). Table 1 summarizes biallelic loss frequencies for each of the 77 genes, including the number of cases with biallelic loss, the number of SNPs with biallelic loss in at least three cases, the number of SNPs mapped within the gene and present on the SNP array, and the fraction of the SNPs with biallelic loss among all the SNPs in the gene. Some of the genes shown in Table 1 are known cancer-associated genes (ie, *FOXP1*, *CSMD1*, *CDKN2A/2B, FHIT*, *DLEC1,* and *RARB)*. Genes affected by biallelic loss were relatively rare (77 genes with biallelic loss divided by an estimated 22,775 genes represented on the Affymetrix array equals approximately 0.0034 or 0.34 %), and far less common than LOH or copy number alterations in ESCC. However, such alterations could be more severe and consequently might have greater impact on tumorigenesis.

**Table 1:** Description of genes with frequent biallelic loss in ESCC^1^

Gene no.	Gene name	Cytoband	No. cases with biallelic loss	No. SNPs with biallelic loss^2^	No. SNPs in gene	Fraction of SNPs in gene with biallelic loss
on 2q						
1	*SPAG16*	2q34	8	7	164	0.04
on 3p						
2	*CADM2*	3p12.1	9	6	44	0.14
3	*CNTN3*	3p12.3	11	9	56	0.16
4	*ROBO1*	3p12.3	9	6	60	0.10
5	*ROBO2*	3p12.3	11	18	93	0.19
6	*EIF4E3*	3p13	9	6	16	0.38
7	*ADAMTS9*	3p14.1	10	10	52	0.19
8	*FAM19A1*	3p14.1	11	15	128	0.12
9	*FOXP1*	3p14.1	12	10	104	0.10
10	*MAGI1*	3p14.1	10	12	192	0.06
11	*PRICKLE2*	3p14.1	9	7	56	0.13
12	*SUCLG2*	3p14.1	10	13	66	0.20
13	*CADPS*	3p14.2	10	11	137	0.08
14	*FHIT*	3p14.2	12	31	345	0.09
15	*PTPRG*	3p14.2	10	28	199	0.14
16	*CACNA2D3*	3p14.2-p21.1	11	22	246	0.09
17	*ERC2*	3p14.3	9	8	211	0.04
18	*PBRM1*	3p21.1	7	8	19	0.42
19	*CACNA2D2*	3p21.31	10	9	17	0.53
20	*DOCK3*	3p21.31	7	8	57	0.14
21	*MYRIP*	3p22.1	9	8	66	0.12
22	*TRAK1*	3p22.1	9	9	39	0.23
23	*DLEC1*	3p22.2	10	5	12	0.42
24	*ITGA9*	3p22.2	11	14	101	0.14
25	*SLC22A14*	3p22.2	8	7	11	0.64
26	*GPD1L*	3p22.3	9	5	14	0.36
27	*OSBPL10*	3p23	9	6	63	0.10
28	*LRRC3B*	3p24.1	7	5	12	0.42
29	*RBMS3*	3p24.1	12	10	166	0.06
30	*RARB*	3p24.2	8	5	42	0.12
31	*THRB*	3p24.2	9	9	84	0.11
32	*RFTN1*	3p24.3	11	13	54	0.24
33	*UBE2E2*	3p24.3	10	9	55	0.16
34	*ZNF385D*	3p24.3	12	21	108	0.19
35	*SLC6A6*	3p25.1	8	5	14	0.36
36	*ATP2B2*	3p25.3	9	10	108	0.09
37	*SRGAP3*	3p25.3	9	12	97	0.12
38	*GRM7*	3p26.1	11	38	276	0.14
39	*LMCD1*	3p26.1	9	5	36	0.14
40	*ITPR1*	3p26.2	11	16	121	0.13
41	*CHL1*	3p26.3	7	6	78	0.08
42	*CNTN4*	3p26.3	10	11	163	0.07
43	*CNTN6*	3p26.3	8	6	111	0.05
on 4p & 4q						
44	*PCDH7*	4p15.1	11	6	58	0.10
45	*KCNIP4*	4p15.31	10	22	338	0.07
46	*LDB2*	4p15.32	7	5	103	0.05
47	*EVC*	4p16.1	8	5	41	0.12
48	*EVC2*	4p16.1	6	5	45	0.12
49	*SORCS2*	4p16.1	6	5	115	0.04
50	*WDR1*	4p16.1	7	5	12	0.42
51	*FSTL5*	4q32.2	11	10	126	0.08
52	*GALNT17*	4q34.1	7	6	187	0.03
on 5q						
53	*RGS7BP*	5q12.3	8	5	37	0.14
54	*IQGAP2*	5q13.3	8	8	87	0.09
55	*SV2C*	5q13.3	7	5	67	0.07
56	*GPR98*	5q14.3	5	6	95	0.06
57	*MCTP1*	5q15	6	5	98	0.05
58	*PPP2R2B*	5q32	7	8	114	0.07
59	*CYFIP2*	5q33.3	7	6	41	0.15
60	*DOCK2*	5q35.1	6	6	166	0.04
61	*SLIT3*	5q35.1	7	5	221	0.02
62	*COL23A1*	5q35.3	9	8	68	0.12
on 8p						
63	*CSMD1*	8p23.2	7	4	153	0.03
on 9p						
64	*LNGCO2*	9p21.1	9	6	140	0.04
65	*CDKN2A/2B*	9p21.1	8	3	6	0.50
66	*KIAA1797*	9p21.3	5	5	57	0.09
67	*MTAP*	9p21.3	8	4	17	0.24
68	*ADAMTSL1*	9p22.1	9	7	108	0.06
69	*C9orf138*	9p22.1	7	5	37	0.14
70	*BNC2*	9p22.2-p22.3	8	7	99	0.07
71	*JMJD2C*	9p24.1	9	6	151	0.04
72	*PTPRD*	9p24.1	9	17	199	0.09
73	*UHRF2*	9P24.1	5	6	19	0.32
74	*GLIS3*	9p24.2	7	5	155	0.03
75	*ANKRD15*	9P24.3	6	7	103	0.07
76	*DOCK8*	9p24.3	7	5	65	0.08
77	*SMARCA2*	9p24.3	6	5	63	0.08

^1^Genes listed in order by chromosome location

^2^No. SNPs with biallelic loss in 3 or more cases

We also examined our ESCC cases by the frequency of biallelic loss frequency among the 7484 SNPs in the 77 genes with biallelic loss (Table 2). Fourteen cases had at least 100 SNPs with biallelic loss and were termed “higher biallelic loss cases”, whereas the remaining 16 cases had fewer than 100 SNPs with biallelic loss and were called “lower biallelic loss cases.” Table 2 summarizes DNA changes (biallelic loss, LOH, and DNA copy number alterations) among the 7848 SNPs in the 77 genes with biallelic loss for each of the 30 ESCC cases. The number of SNPs affected by LOH and copy number alterations varied widely among cases. The number of SNPs with biallelic loss was highly correlated with both the number of SNPs with LOH (*r* = 0.92, *p* = 4.41E-13) and the number of SNPs with copy number loss (*r* = 0.97, *p* = 3.94E-19), but was not significantly correlated with either the number of SNPs with copy number gain (*r* = 0.21, *p* = 0.26) or the number of SNPs with copy number neutral LOH (*r* = 0.18, *p* = 0.34).

**Table 2:** Description of DNA alterations in SNPs found in genes with frequent biallelic loss among ESCC cases^1^

Row no.	Case no.	No. SNPs with biallelic loss	No. SNPs with LOH	No. SNPs with CN loss	No. SNPs with CN gain	No. SNPs with CN neutral LOH
1	E24	1304	479	5173	0	9
2	E4	1251	193	3686	0	166
3	E30	1082	799	4449	0	15
4	E27	895	436	3858	0	114
5	E14	796	338	3845	0	266
6	E26	769	928	4115	0	310
7	E6	762	114	2244	107	173
8	E23	720	583	3870	0	21
9	E29	707	185	3384	191	164
10	E22	657	661	2722	133	217
11	E17	435	272	2735	428	340
12	E10	283	60	1474	746	41
13	E13	213	71	1411	824	202
14	E8	170	44	1341	118	0
15	E18	91	8	1089	282	137
16	E1	90	69	383	0	956
17	E11	70	126	343	0	1193
18	E28	53	9	1063	0	31
19	E25	47	4	370	111	426
20	E9	29	1	538	67	29
21	E16	29	0	164	0	64
22	E2	21	0	245	0	81
23	E7	17	1	108	0	95
24	E21	6	1	16	0	673
25	E3	1	0	26	0	72
26	E5	0	0	0	0	45
27	E12	0	0	18	0	13
28	E15	0	0	4	0	14
29	E19	0	0	11	0	23
30	E20	0	0	0	0	53

^1^Cases listed in order by no. SNPs with biallelic loss; cases with 100+ SNPs with biallelic loss = ‘higher loss cases’, <100 = ‘lower loss cases’

We also checked for microdeletions or biallelic loss regions on chromosomes (eg, 3p14) caused by continuous SNPs with biallelic loss, but were unable to identify any.

### Biallelic loss and expression of mRNA and miRNA

**Fig. 1:**
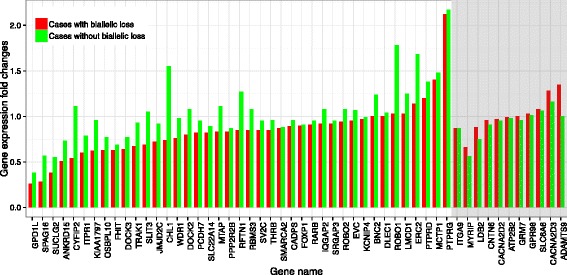
mRNA expression by biallelic loss status for 52 genes in ESCC. X-axis: names of 52 genes present on the Affymetrix HU 133 A chip with a signal present in both tumor and matched normal tissues; Y-axis: gene expression fold changes (median log 2 ratio) in cases with biallelic loss (*red*) and in cases without biallelic loss (*green*)

Among the 77 genes with biallelic loss, 52 had probes represented on the Affymetrix Hu 133 array with signals in both tumor and normal tissues. We found that 41 of these 52 genes (79 %) had lower mRNA expression levels in cases with biallelic loss than cases with no biallelic loss (the 41 genes shown in the unshaded area on the left in Fig. 1), including eight genes in which mRNA expression was statistically significantly lower (Additional file 2: Table S1). For example, the mean fold change in *MTAP* expression was 0.83 in cases with biallelic loss versus 1.11 in cases with no biallelic loss (*P* = 0.009). Eleven genes had expression levels that were the same or higher in cases with biallelic loss than cases with no loss (the 11 genes in the shaded area on the right in Fig. 1). As an example, the median fold change for expression of *ADAMTS9* was 1.35 in cases with biallelic loss compared to 1.0 in cases without biallelic loss (Additional file 2: Table S1). Although expression differences observed were modest, these results suggest that biallelic loss appeared to influence gene expression.

Expression of miRNAs by biallelic loss status is shown in Additional file 3: Table S2. The ratio of miRNA expression in target genes with biallelic loss (versus without) was greater than one for 34 of 60 (57 %) miRNAs and less than one in 26 of 60 (43 %).

### miRNA expression and target gene expression in genes with frequent biallelic loss

There were a total of 664 miRNAs on the ABI Chips A & B in our analysis. Two hundred sixty-eight miRNAs were excluded from further analysis because of inadequate data (ie, signal was present only in tumor or only in normal, or signal was absent altogether because of tissue specificity), leaving 396 miRNAs that showed signals in both the tumor and the normal tissues in at least 10 % of the 30 ESCC cases (ie, at least three cases) for our analyses (Additional file 4: Table S3 and Additional file 5: Table S4). We checked the conserved miRNA targets in the 3′ UTRs for the 52 genes with mRNA results using http://www.targetscan.org/ and found 44 genes that could be targeted by one or more of the miRNAs present on the ABI miRNA array. After further filtering (ie, seven genes were not targeted by miRNAs on our array, and two genes had miRNA signals in less than three cases), we had data available to analyze the relation between the expression of 70 miRNAs (58 targeted just one gene and 12 targeted multiple genes) and 35 gene targets in our 30 ESCC cases (Additional file 6: Table S5). We found a relatively wide range of miRNA expression levels among these gene targets (tumor:normal miRNA fold change median = 1.62, range 0.08 to 5.27; Additional file 7: Table S6). Overall, in the 35 genes with biallelic loss that were evaluable, miRNA expression levels were more often elevated (fold change > 1.0) than mRNA expression levels (69 % versus 14 %, respectively). When miRNA and mRNA were examined together, expression levels of 41 (of 70) miRNAs were elevated (fold change > 1.0) while their target gene expression levels were reduced (fold change < 1.0). Examples of interesting miRNA-target gene pairs that showed this pattern were: expression of miR-205 was 3.11-fold and its target gene *ADAMTS9* expression was 0.87-fold; miR-124 was 3.79-fold and its target gene *SUCLG2* was 0.42-fold; and expression of miR-183 was 3.01-fold while its target *FOXP1* was 0.76-fold. Conversely, 17 miRNAs showed reduced expression in both the miRNA and its target gene. An illustrative example of this relation is the 0.17-fold expression change for miR-133b0.85-fold in its target gene *IQGAP* expression. Taken together, our results indicate that miRNA expression levels varied widely. Increased miRNA most often was associated with reduced target gene expression, but reduced target gene expression was also frequently seen with reduced miRNA in ESCC.

## Discussion

In the present study, we took an integrative approach by evaluating genes in relation to biallelic loss and expression of both mRNA and miRNA in ESCC cases using profiling data generated from arrays. We made several observations from our evaluation of these data. First, biallelic loss was relatively uncommon, but when it occurred it was concentrated in four chromosome arms, namely, 3p, 9p, 5q, and 4p. Second, biallelic loss appeared to affect gene expression; nearly 80 % of genes in cases with biallelic loss showed reduced mRNA expression compared to those without loss. Third, although the relation was less clear than for mRNA, biallelic loss also appeared to affect miRNA expression. More informative future studies will need larger sample sizes so as to have more heterozygous SNPs for evaluation, and appropriate coverage of promoter regions, to confirm and expand our findings here regarding relations between SNPs with biallelic loss and expression of mRNA and miRNA.

Distributions of miRNA expressions were generally higher in cases with biallelic loss than in cases without loss. Of 60 miRNA-target gene pairs, 34 pairs (57 %) had higher miRNA expression with biallelic loss than without, while 26 pairs (43 %) had lower miRNA expression. Finally, the effect of biallelic loss on the relation between miRNA and mRNA expression was complex. Biallelic loss was most commonly associated with a pattern of elevated miRNA and reduced mRNA (43 %), but a pattern of both reduced miRNA and mRNA was also common (35 %).

In our study, 77 genes showed biallelic loss and half of these genes were located on chromosome 3p, our most common site of loss. The frequency of biallelic loss among the 42 genes on chromosome 3p ranged from 5 % to 64 % (Table 1). This region includes several tumor suppressor genes (eg, *ROBO1*, *FOXP1, FHIT*). Our previous studies also showed high frequency of LOH on chromosome 3p in ESCC [5]. Taken together, biallelic loss, similar to LOH, appears to play a role in the stability of chromosome 3p in ESCC. Although most SNPs that show biallelic loss and/or LOH are located in the non-coding regions of these genes, they may exert their effects via gene expression. For example, biallelic loss of *CHL1* (3p26.3) affected six of 78 SNPs, and the expression level of *CHL1* was reduced in cases with biallelic loss but elevated in cases without biallelic loss (Additional file 2: Table S1), while miRNA-10a and miRNA-10b expression levels, which both target *CHL1*, were higher in cases with biallelic loss than those without (Additional file 3: Table S2). Interestingly, a previous study showed that miR-10b may play a causal role in inducing metastatic behavior [22]. We note that expression levels for some genes did not show big differences by biallelic loss status, or even higher expression levels in cases with biallelic loss than those without. One potential explanation for this finding is epigenetic alterations such as DNA methylation changes.

Our results indicate that ESCC tumors may be divided into two groups: those with high and those with low levels of genome instability as assessed by the number of SNPs with biallelic loss (Table 2), suggesting that the genetic stability is variable, even among patients from a seemingly homogeneous population with extraordinarily high rates of esophageal cancer. Our results also show that both miRNA and mRNA levels varied widely despite the fact that the esophageal cancer patients studied here were similar in many important ways (eg, from the same geography, had the same tumor histology, and had similar clinical characteristics such as stage) [5], suggesting that ESCC is incredibly complex and that there is significant heterogeneity among ESCC patients. This is likely one reason why tumors that appear similar can progress and respond to therapy in dramatically different ways. New insights gained from better understanding of case/tumor heterogeneity should be useful for predicting response to therapy [23, 24].

Although several studies have evaluated biallelic loss/homozygous deletion within specific genes in tumors (e.g., *CDKN2A* [25]), including ESCC [26], using techniques such as FISH, only a few prior reports have used genome-wide techniques such as SNP or comparative genomic hybridization (CGH) arrays to agnostically assess homozygous deletions in tumor cell lines or tissues. Cancers evaluated for biallelic loss with array technology include prostate (SNP array) [25, 27], B-cell lymphomas (CGH array) [28], and B-cell chronic lymphocytic leukemia (SNP array) [29]. Among the largest of these studies, Guichard et al. used CGH arrays and recently reported that 40 % of 125 hepatocellular carcinomas had homozygous deletions [30]. Twelve regions were recurrently altered, including most frequently loci at *CDKN2A-CDKN2B* (6.4 %), *AXIN1* (3.2 %), and *IRF2* (3.2 %). To the best of our best knowledge, our study is the first report of biallelic loss identified in genes in ESCC cases using array technology.

We note that there are several limitations in the current study, most notably our small sample size. In addition, most of the SNPs identified with biallelic loss were in introns of genes, and our small sample size precluded detailed assessment of interactions among DNA, RNA, and miRNA. A major strength of this study is that all 30 ESCC cases reported were evaluated using the same array platforms and every case had both tumor and normal tissue DNA, RNA, and miRNA profiled using genome-wide methods, so that our comparisons are comprehensive and carefully controlled paired comparisons within the same case.

## Conclusion

In conclusion, our results indicate that biallelic loss in ESCC is uncommon, but when it occurs it is localized to a few specific chromosomal regions and appears to influence mRNA expression of affected genes, leading to complex patterns of expression of miRNA and target genes in ESCC patients.

## Additional files

Additional file 1: Figure S1. Flow diagram of laboratory analyses. (PPT 106 kb) Additional file 2: Table S1. Average tumor:normal mRNA expression fold changes by biallelic loss status for 52 genes in ESCC cases. (XLS 29 kb) Additional file 3: Table S2. Expression of miRNAs (*n* = 60) and targeted genes (*n* = 32) in ESCC cases (*n* = 30) by biallelic loss status. (XLSX 11 kb) Additional file 4: Table S3. Tumor:normal fold change for expression of 396 miRNAs in 30 ESCC cases. (XLS 283 kb) Additional file 5: Table S4. Number and frequency of dysregulated miRNAs in ESCC cases. (XLS 31 kb) Additional file 6: Table S5. Tumor:normal expression fold changes for miRNAs and 35 target gene mRNAs in 30 ESCC cases. (XLS 120 kb) Additional file 7: Table S6. miRNA and targeted gene tumor:normal expression fold changes (70 miRNAs, 35 genes, 30 ESCC cases). (XLS 30 kb)

## Abbreviations

SNP: Single nucleotide polymorphism
ESCC: Esophageal squamous cell carcinoma
FC: Fold change

## References

[1] Parkin DM, Bray F, Ferlay J, Pisani P (2005). Global cancer statistics, 2002. CA Cancer J Clin.

[2] Yang L, Parkin DM, Ferlay J, Li L, Chen Y (2005). Estimates of cancer incidence in China for 2000 and projections for 2005. Cancer Epidemiol Biomarkers Prev.

[3] Jemal A, Siegel R, Xu J, Ward E (2010). Cancer statistics, 2010. CA Cancer J Clin.

[4] Nancarrow DJ, Handoko HY, Smithers BM, Gotley DC, Drew PA, Watson DI, Clouston AD, Hayward NK, Whiteman DC (2008). Genome-wide copy number analysis in esophageal adenocarcinoma using high-density single-nucleotide polymorphism arrays. Cancer Res.

[5] Hu N, Wang C, Ng D, Clifford R, Yang HH, Tang ZZ, Wang QH, Han XY, Giffen C, Goldstein AM, Taylor PR, Lee MP (2009). Genomic characterization of esophageal squamous cell carcinoma from a high-risk population in China. Cancer Res.

[6] Hu N, Clifford RJ, Yang HH, Wang C, Goldstein AM, Ding T, Taylor PR, Lee MP (2010). Genome wide analysis of DNA copy number neutral loss of heterozygosity (CNNLOH) and its relation to gene expression in esophageal squamous cell carcinoma. BMC Genomics.

[7] Hu N, Huang J, Emmert-Buck MR, Tang ZZ, Roth MJ, Wang C, Dawsey SM, Li G, Li WJ, Wang QH, Han XY, Ding T, Giffen C, Goldstein AM, Taylor PR (2001). Frequent inactivation of the TP53 gene in esophageal squamous cell carcinoma from a high-risk population in China. Clin Cancer Res.

[8] Hu N, Wang C, Su H, Li WJ, Emmert-Buck MR, Li G, Roth MJ, Tang ZZ, Lu N, Giffen C, Albert PS, Taylor PR, Goldstein AM (2004). High frequency of CDKN2A alterations in esophageal squamous cell carcinoma from a high-risk Chinese population. Genes Chromosomes Cancer.

[9] Hu N, Wang C, Han XY, He LJ, Tang ZZ, Giffen CA, Emmert-Buck MR, Goldstein AM, Taylor PR (2004). Evaluation of BRCA2 in the genetic susceptibility of familial esophageal cancer. Oncogene.

[10] Su H, Hu N, Yang HH, Wang C, Takikita M, Wang QH, Giffen C, Clifford R, Hewitt SM, Shou JZ, Goldstein AM, Lee MP, Taylor PR (2011). Global gene expression profiling and validation in esophageal squamous cell carcinoma and its association with clinical phenotypes. Clin Cancer Res.

[11] Tamoto E, Tada M, Murakawa K, Takada M, Shindo G, Teramoto K, Matsunaga A, Komuro K, Kanai M, Kawakami A, Fujiwara Y, Kobayashi N, Shirata K, Nishimura N, Okushiba S, Kondo S, Hamada J, Yoshiki T, Moriuchi T, Katoh H (2004). Gene-expression profile changes correlated with tumor progression and lymph node metastasis in esophageal cancer. Clin Cancer Res.

[12] Ishibashi Y, Hanyu N, Nakada K, Suzuki Y, Yamamoto T, Yanaga K, Ohkawa K, Hashimoto N, Nakajima T, Saito H, Matsushima M, Urashima M (2003). Profiling gene expression ratios of paired cancerous and normal tissue predicts relapse of esophageal squamous cell carcinoma. Cancer Res.

[13] Hu YC, Lam KY, Law S, Wong J, Srivastava G (2001). Profiling of differentially expressed cancer-related genes in esophageal squamous cell carcinoma (ESCC) using human cancer cDNA arrays: overexpression of oncogene MET correlates with tumor differentiation in ESCC. Clin Cancer Res.

[14] Kan T, Shimada Y, Sato F, Maeda M, Kawabe A, Kaganoi J, Itami A, Yamasaki S, Imamura M (2001). Gene expression profiling in human esophageal cancers using cDNA microarray. Biochem Biophys Res Commun.

[15] Su H, Hu N, Shih J, Hu Y, Wang QH, Chuang EY, Roth MJ, Wang C, Goldstein AM, Ding T, Dawsey SM, Giffen C, Emmert-Buck MR, Taylor PR (2003). Gene expression analysis of esophageal squamous cell carcinoma reveals consistent molecular profiles related to a family history of upper gastrointestinal cancer. Cancer Res.

[16] Esquela-Kerscher A, Slack FJ (2006). Oncomirs - microRNAs with a role in cancer. Nat Rev Cancer.

[17] Patnaik SK, Mallick R, Yendamuri S (2010). MicroRNAs and esophageal cancer. J Gastrointest Oncol.

[18] David S, Meltzer SJ (2011). MicroRNA involvement in esophageal carcinogenesis. Curr Opin Pharmacol.

[19] Guo Y, Chen Z, Zhang L, Zhou F, Shi S, Feng X, Li B, Meng X, Ma X, Luo M, Shao K, Li N, Qiu B, Mitchelson K, Cheng J, He J (2008). Distinctive microRNA profiles relating to patient survival in esophageal squamous cell carcinoma. Cancer Res.

[20] Mathe EA, Nguyen GH, Bowman ED, Zhao Y, Budhu A, Schetter AJ, Braun R, Reimers M, Kumamoto K, Hughes D, Altorki NK, Casson AG, Liu CG, Wang XW, Yanaihara N, Hagiwara N, Dannenberg AJ, Miyashita M, Croce CM, Harris CC (2009). MicroRNA expression in squamous cell carcinoma and adenocarcinoma of the esophagus: associations with survival. Clin Cancer Res.

[21] Gao Y, Hu N, Han XY, Ding T, Giffen C, Goldstein AM, Taylor PR (2011). Risk factors for esophagel and gastric cancers in Shanxi Province, China: A case–control study. Cancer Epidemiol.

[22] Ma L, Reinhardt F, Pan E, Soutschek J, Bhat B, Marcusson EG, Teruya-Feldstein J, Bell GW, Weinberg RA (2010). Therapeutic silencing of miR-10b inhibits metastasis in a mouse mammary tumor model. Nat Biotechnol.

[23] Gray JW, Collins C (2000). Genome changes and gene expression in human solid tumors. Carcinogenesis.

[24] Lord CJ, Ashworth A (2012). The DNA damage response and cancer therapy. Nature.

[25] Sulong S, Moorman AV, Irving JA, Strefford JC, Konn ZJ, Case MC, Minto L, Barber KE, Parker H, Wright SL, Stewart AR, Bailey S, Bown NP, Hall AG, Harrison CJ (2009). A comprehensive analysis of the CDKN2A gene in childhood acute lymphoblastic leukemia reveals genomic deletion, copy number neutral loss of heterozygosity, and association with specific cytogenetic subgroups. Blood.

[26] Xing EP, Nie Y, Song Y, Yang GY, Cai YC, Wang LD, Yang CS (1999). Mechanisms of inactivation of p14ARF, p15INK4b, and p16INK4a genes in human esophageal squamous cell carcinoma. Clin Cancer Res.

[27] Liu W, Xie CC, Zhu Y, Li T, Sun J, Cheng Y, Ewing CM, Dalrymple S, Turner AR, Sun J, Isaacs JT, Chang BL, Zheng SL, Isaacs WB, Xu J (2008). Homozygous deletions and recurrent amplifications implicate new genes involved in prostate cancer. Neoplasia.

[28] Mestre-Escorihuela C, Rubio-Moscardo F, Richter JA, Siebert R, Climent J, Fresquet V, Beltran E, Agirre X, Marugan I, Marin M, Rosenwald A, Sugimoto KJ, Wheat LM, Karran EL, Garcia JF, Sanchez L, Prosper F, Staudt LM, Pinkel D, Dyer MJ, Martinez-Climent JA (2007). Homozygous deletions localize novel tumor suppressor genes in B-cell lymphomas. Blood.

[29] Mosca L, Fabris S, Lionetti M, Todoerti K, Agnelli L, Morabito F, Cutrona G, Andronache A, Matis S, Ferrari F, Gentile M, Spriano M, Callea V, Festini G, Molica S, Deliliers GL, Bicciato S, Ferrarini M, Neri A (2010). Integrative genomics analyses reveal molecularly distinct subgroups of B-cell chronic lymphocytic leukemia patients with 13q14 deletion. Clin Cancer Res.

[30] Guichard C, Amaddeo G, Imbeaud S, Ladeiro Y, Pelletier L, Maad IB, Calderaro J, Bioulac-Sage P, Letexier M, Degos F, Clement B, Balabaud C, Chevet E, Laurent A, Couchy G, Letouze E, Calvo F, Zucman-Rossi J (2012). Integrated analysis of somatic mutations and focal copy-number changes identifies key genes and pathways in hepatocellular carcinoma. Nat Genet.

