# Supplementary material for: Integrative genomics analysis of genes with biallelic loss and its relation to the expression of mRNA and micro-RNA in esophageal squamous cell carcinoma
Source: BMC Genomics. 2015 Sep 26;16:732. doi: 10.1186/s12864-015-1919-0 (PMC4584010; doi:10.1186/s12864-015-1919-0)
Supplement: Additional file 1: Figure S1. — Flow diagram of laboratory analyses. (PPT 106 kb) [file 12864_2015_1919_MOESM1_ESM.ppt]

## Slide 1
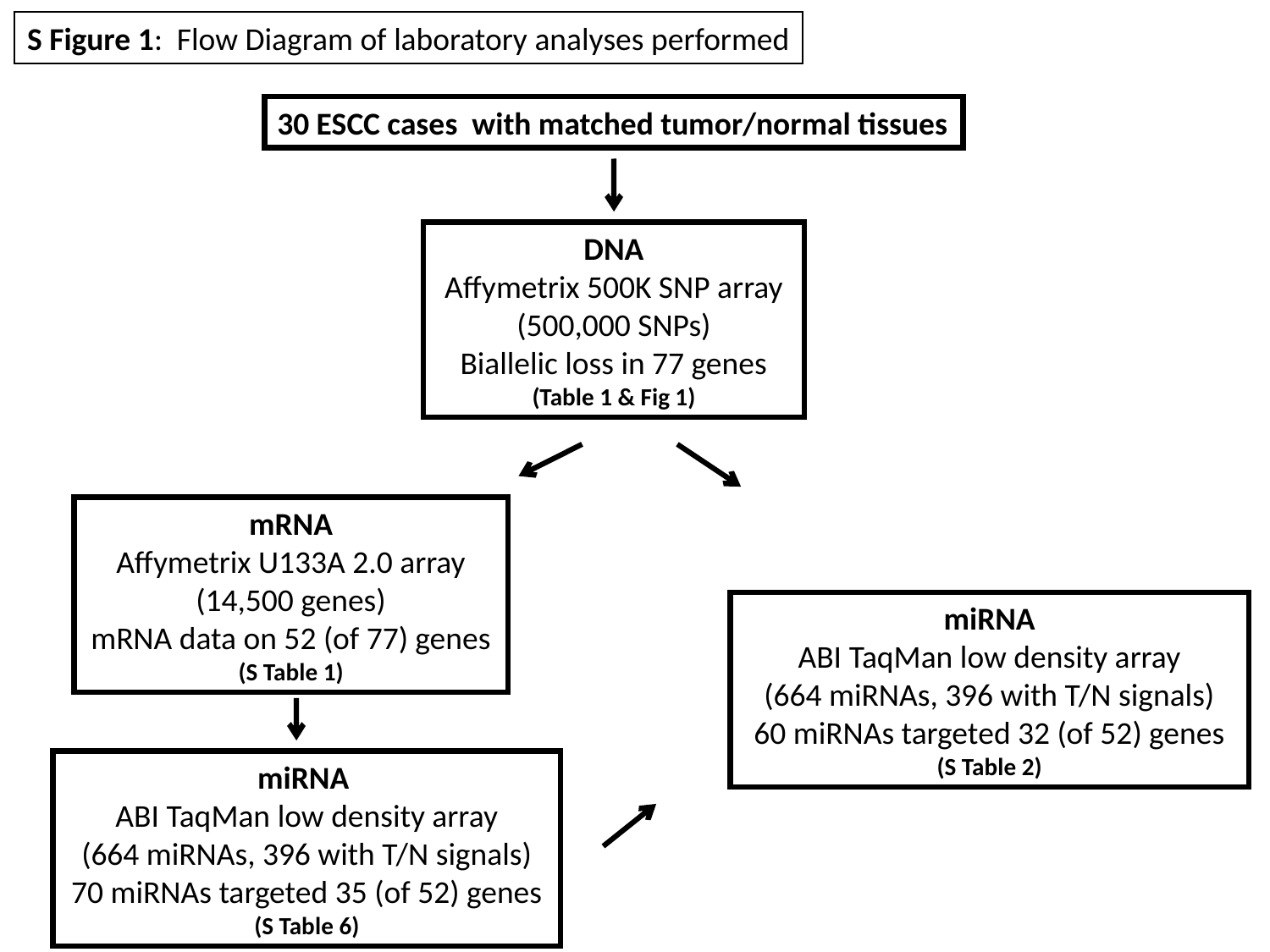

S Figure 1: Flow Diagram of laboratory analyses performed
30 ESCC cases with matched tumor/normal tissues
DNA
Affymetrix 500K SNP array
(500,000 SNPs)
Biallelic loss in 77 genes
(Table 1 & Fig 1)
mRNA
Affymetrix U133A 2.0 array
(14,500 genes)
mRNA data on 52 (of 77) genes
(S Table 1)
miRNA
ABI TaqMan low density array
(664 miRNAs, 396 with T/N signals)
60 miRNAs targeted 32 (of 52) genes
(S Table 2)
miRNA
ABI TaqMan low density array
(664 miRNAs, 396 with T/N signals)
70 miRNAs targeted 35 (of 52) genes
(S Table 6)
